# Selection of Behavior Change Techniques for Asthma Medication Adherence Apps: Evidence-Based Design Study

**DOI:** 10.2196/49348

**Published:** 2025-09-23

**Authors:** Alison J Wright, Jeremy Holland, Iain Simpson, Samantha Walker, Naomi Bennett-Steele, John Weinman

**Affiliations:** 1 Institute of Pharmaceutical Science, King's College London, London, United Kingdom; 2 Healthcare Programmes, Centre for Business Innovation, 18 Sedley Taylor Road, Cambridge, CB2 8PW, United Kingdom, 44 7796265994; 3 Pharmaceutical Drug Delivery, Phillips Medisize, Cambridge, United Kingdom; 4 Research and Innovation, Asthma + Lung UK, London, United Kingdom

**Keywords:** medication adherence, patient adherence, behavior change, asthma, apps for medication adherence, medication, app, behavior, patient outcome, app design, design, acceptability, practicability, decision-making, decision

## Abstract

**Background:**

Poor medication adherence is a widespread issue that causes adverse patient outcomes and is expensive for all aspects of the health care system. Developing cost-effective and scalable interventions to promote medication adherence is a key goal. Mobile apps hold promise as a mode of delivery for adherence interventions, but app design rarely takes into account the behavioral influences on nonadherence with sufficient rigor. As a result, apps may not realize their full potential in enhancing adherence. Medication nonadherence is common among adults prescribed preventer inhalers for asthma and has a variety of influences, creating a need to identify what components behavior change technique (BCT) apps should include to effectively tackle each influence.

**Objective:**

This study aimed to identify the most acceptable and practicable BCTs to include in a medication adherence app targeting factors that influence preventer inhaler adherence in adults with asthma.

**Methods:**

Key influences on preventer inhaler adherence in adults with asthma were identified based on reviews of peer-reviewed and gray literature and domain expert knowledge. These influences were then mapped to a published set of 26 mechanisms of action (MoAs) of behavior change interventions. Next, candidate BCTs to change each MoA were identified using the Theory and Techniques tool, a web-based resource that reflects almost 100 expert behavioral scientists’ consensus about which BCTs are most likely to change particular MoAs. Finally, candidate BCTs were filtered by considering their potential acceptability and practicability.

**Results:**

A total of 31 influences on preventer inhaler adherence were identified and coded to 15/26 of the influences on behavior listed by the Theory and Techniques tool. The initial mapping of influences on behavior to candidate BCTs to change those influences identified 41 candidate BCTs. After considering the potential acceptability and practicability of the candidate BCTs, the number of BCTs suggested for inclusion was reduced to 24.

**Conclusions:**

Using an evidence-based approach, this study identified 24 BCTs that may be particularly useful to include in apps promoting adherence to preventer inhalers in order to target particular influences on adherence. The list can be used by app developers to improve the quality of adherence behavior change support that their apps provide or by health care decision-makers to identify which apps contain elements addressing a range of adherence difficulties.

## Introduction

### Background

The World Health Organization estimates that 30%‐50% of patients do not take their long-term medication as prescribed [1]. Medication nonadherence in long-term conditions is associated with reduced quality of life, poorer clinical outcomes, increased risk of premature death, and increased use of health care services. Estimates suggest that nonadherence contributes to nearly 200,000 premature deaths per year and costs European governments €125 billion (1 €=US $1.15) annually in excess health care services [2]. Taking medication as prescribed is a behavior, and so it is helpful to use a behavioral science perspective to understand and promote it. While there are a number of approaches to behavior change intervention design, many share the approach of identifying modifiable influences on the target behavior and then identifying appropriate intervention components to change these modifiable influences.

Considerable effort has been devoted to the development and evaluation of interventions to enhance medication adherence [3]. Complex, carefully designed interventions have been shown to improve both adherence and health outcomes [4,5]. However, the difficulty is making these interventions sufficiently cost-effective and scalable. Moreover, adherence interventions delivered by health care professionals may not be offered at the times or in the locations most convenient to people with the health problem in question. In contrast, the pervasiveness of smartphones in high-income countries makes the potential of apps for providing convenient, lower-cost, and scalable yet personalized medication adherence interventions very appealing in this context. A recent meta-analysis of randomized controlled trials found that app-based medication adherence interventions enhanced self-reported adherence [6], but firm conclusions were hard to make due to the small number of included studies, the heterogeneity of study populations, and variation in how adherence was measured in different trials.

Medication adherence is a behavior, and so interventions to target adherence will benefit from building on behavioral science evidence. Based on the literature, behavioral scientists developed a taxonomy of 93 behavior change techniques (BCTs), defined as the smallest self-contained components of an intervention aimed at changing behavior [7]. Examples of BCTs include “action planning” (making a detailed plan for the performance of the behavior, which includes at least one of context, frequency, duration, or intensity) and “social comparison” (drawing attention to other people’s behavior and comparing their behavior with the person’s own behavior). A review of 166 apps targeting medication adherence [8] discovered that only 12 of over 90 possible BCTs were found in at least one of the apps. The most commonly used BCTs were “action planning” and “prompts or cues,” both of which target developing a medication-taking routine and reducing the chances of forgetting to take a dose of medication. However, medication adherence is influenced by many factors, of which having a routine and remembering to take the medication are only 2 [9,10]. Apps are likely to be more effective when they target the full range of key influences on medication adherence behavior. A related issue is that many apps are not tailored to the specific causes of medication nonadherence for individual users. For example, an app providing simple reminders to take medication is unlikely to benefit a patient who is concerned about potential side effects. Similarly, providing information about medication benefits is unlikely to make a difference to the adherence of a patient who is already motivated to take their medicine but is nonadherent due to a lack of a routine for doing so.

Where apps do attempt to tackle specific influences on medication adherence, their developers face several challenges. First, they need to integrate a method of assessing the influences that are most relevant to each app user’s adherence. Second, app designers may have difficulty applying evidence as to which of these BCTs are most likely to change these influences, due to the expertise required to assess and interpret this evidence. A further issue in selecting intervention components is that some BCTs may not be acceptable to app users or practical for individuals to use in their daily lives. Therefore, we need a way to identify what BCTs a medication adherence app could usefully contain based on evidence about the causes of nonadherence and considering the likely acceptability and practicality of deploying these BCTs in an app. Identifying these BCTs gives app developers and designers a set of potential intervention components to work with.

This study originated from a working group of the Centre for Business Innovation Medical Adherence and Digital Health consortium, a European interdisciplinary and primarily industrial grouping, which meets regularly to consider key issues around the role of digital health in improving medication adherence. For the present piece of work, the Centre for Business Innovation collaborated with Asthma UK (now Asthma+ Lung UK) to map out the behavioral factors influencing adherence to preventer medication for adults with asthma and apply a rigorous approach to identify the BCTs most relevant to promoting medication adherence for people with asthma. Asthma is a common long-term condition, affecting an estimated 260 million people worldwide in 2019 [11]. Each year, there are 455,000 asthma-related deaths, two-thirds of which are preventable [12]. For the vast majority of people with asthma, the regular use of “preventer” inhalers is recommended to avoid or reduce experiencing asthma attacks. However, nonadherence is common, with observed adherence rates between 22% and 63% [13]. Nonadherence is associated with poorer symptom control, reduced quality of life, and an increased risk of emergency health care use and even mortality [13]. Delivering medication adherence support via an app may be particularly helpful for adults with asthma, many of whom are of working age and so may find attending clinic appointments for adherence support inconvenient.

Nonadherence to preventer inhalers has a variety of causes, such as not perceiving asthma inhalers as necessary, being concerned about side effects, or having difficulties accessing medication, which vary for different people with asthma [14,15]. Therefore, there is a need to identify the optimal BCTs to tackle specific influences on preventer inhaler nonadherence via an app-based intervention. Medication adherence apps have the potential to be tailored to tackle the barriers most relevant to a particular person with asthma. However, a small systematic review of app-based interventions targeting medication adherence in people with asthma found only 9 BCTs in the 4 evaluated apps [16]. Another recent study examined the extent to which 23 asthma apps contained BCTs alongside rating their quality in terms of 5 user experience domains [17]. The number of BCTs in each app ranged from 1 to 11, and those rated as higher quality contained more BCTs. However, these BCTs were not linked to specific influences or causes of nonadherence in any logical way. A second recent study [18] examined the functionality and quality of asthma mHealth apps, but focused only on the broad categories of BCTs that are included in the Mobile App Rating Scale. In the 53 apps they reviewed, the most commonly used BCTs were in the broad categories of “information or education” (62% of apps) and “self-monitoring or tracking” (72% of apps). However, there was no assessment of whether the included BCTs were appropriately linked to targeted influences on asthma self-management. The process of identifying BCTs to tackle different influences on nonadherence to preventer inhalers serves as a good exemplar of the process of identifying BCTs to feature in apps promoting adherence to medication in other conditions.

### Objectives

The purpose of this article is to describe and report the outcomes of a process to identify which BCTs could be included in a medication adherence app to promote adherence to preventer inhalers in people with asthma.

The article also serves as a case study of using behavioral science evidence to move from understanding the influences on nonadherence to deriving a set of BCTs that can be used in a medication adherence app.

## Methods

### Overview

To identify which BCTs might be usefully included in an adherence app, we used a 4-stage process:

1. Identify modifiable influences on adherence.
2. Map the influences to a published list of mechanisms of action (MoA) of behavior change interventions.
3. Map MoAs to the BCTs, which expert consensus indicated were likely to change those MoAs.
4. Filter BCTs in terms of their likely acceptability and practicability.

We describe each step in more detail in later sections.

### Identifying Influences on Medication Adherence

For any particular health condition and therapy combination, we must first identify the influences on medication adherence. Asthma + Lung UK worked with Public Health England to compile an evidence-based list of barriers and facilitators of preventer inhaler adherence. Please see the multimedia appendix for the details of this process. Two types of sources were used to provide data on the barriers and enablers to the target behavior. The first, identified by Asthma + Lung UK and classified as gray literature, were unpublished reports of findings from qualitative studies on asthma self-management and barriers to self-management behaviors that were commissioned or conducted by Asthma + Lung UK as part of their work on behavioral insights in asthma (these reports are listed in Table S1 in the Multimedia Appendix 1). The second source was a rapid review of barriers and enablers to preventer inhaler adherence reported in the published literature. The rapid review synthesized published, peer-reviewed reviews of either quantitative or qualitative studies that reported barriers or facilitators to asthma self-management behaviors, including medication adherence (please see the Multimedia Appendix 1 for a list of the included reviews). Rapid review methods [19,20] were chosen in order to provide a solid overview of the evidence on barriers and facilitators to adherence in a more timely and less resource-intensive fashion than a full systematic review would have required.

Data pertinent to the influences (barriers and facilitators) to adherence were extracted from the results sections of the gray literature reports and published reviews. Supporting statements for each identified theme were extracted, including quotes from the original participants, if available, or the author’s interpretation/narration of the findings. Text reporting barriers and facilitators was coded using inductive thematic coding by one reviewer. The findings were then checked by another reviewer, and any disagreements were resolved through discussion.

### Mapping Influences to Behavior Change Intervention MOAs

To be able to use expert consensus evidence about which BCTs were best to change particular influences on behavior, we mapped each influence identified in Step 1 to the list of 26 intervention mechanisms of action used in a recent expert consensus project [21-24]. For example, “beliefs about side effects” was mapped to the MoA called “beliefs about consequences” which is defined as “Beliefs about the consequences of a behavior (ie, perceptions about what will be achieved and/or lost by undertaking a behavior, as well as the probability that a behavior will lead to a specific outcome,)” because side effects are a consequence of taking medication. Mapping was conducted by 1 author (AJW). A second author (JW) then independently reviewed the mapping. Any disagreements between the authors were resolved through discussion.

### Map MoAs to BCTs That Expert Consensus Indicates Will Change Each MoA

Ideally, the choice of a BCT to change a particular MoA would be based on evidence showing that the BCT changed the MoA in experimental studies targeting the relevant behavior. However, such evidence does not yet exist for every BCT-MoA-behavior combination that intervention designers might be interested in. Given this, intervention designers may instead have to rely on expert consensus. A recent project [24] triangulated expert consensus on the links between 26 MoAs and 56 BCTs commonly used in behavior change interventions and created a web-based tool that allows users to view which BCTs experts thought were likely to influence particular MoAs. We used this web-based tool to identify what BCT experts thought were linked to each of the MoAs identified in the previous step. For many MoAs, more than one BCT was linked to that MoA. For example, the MoA of “beliefs about consequences” was linked to ten BCTs, such as “provide information about health consequences,” “anticipated regret,” “pros and cons,” “comparative imagining of future outcomes,” and “material incentive for behavior” to name but a few.

### Filter BCTs in Terms of Their Likely Acceptability and Practicability

To reduce the number of candidate BCTs that intervention developers might need to consider, we applied the “acceptability” and “practicability” elements of the APEASE (Acceptability, Practicability, Effectiveness and cost-effectiveness, Affordability, Side effects/safety, and Equity) criteria suggested by West et al and Michie et al [25,26], as a way to select elements of interventions. “Acceptability” concerns how far an intervention component is likely to be acceptable to key stakeholders, including target participants. “Practicability” concerns whether the intervention component can be implemented at scale within the intended context, material, and human resources. “Effectiveness” (and cost-effectiveness) concerns how likely the intervention is to achieve the intended outcomes and be good value for money. “Side effects/safety” concerns whether the intervention poses any safety concerns, while “Equity” refers to whether the intervention might reduce or increase social inequalities.

To apply the APEASE criteria, one has to specify the context in which the behavior change intervention will take place. In this case, the relevant context was that the app should work as a standalone intervention for adults with asthma prescribed preventer inhalers. Although it might be recommended to a patient by a health care professional, the app should not require further health care professional involvement because part of the impetus for creating an app was to have an adherence intervention that did not need considerable input from time-pressed health care professionals. We also specified that the app should not require the patient to have any further technology (eg, a laptop or a way of monitoring lung functioning) beyond their smartphone. Finally, we assumed that the app was to be rolled out in a high-income country where asthma is largely managed in primary care.

Each candidate BCT was appraised by 2 behavioral science experts with expertise in the design of behavior change interventions targeting adherence (AJW and JW). Any disagreements were resolved through discussion between the 2 experts. The experts first considered the practicability of delivering each BCT via an app in the context specified above. For BCTs judged practicable, the experts then considered their potential acceptability to app users. For example, the BCT of “provide information about health consequences” was seen as practicable to deliver within an app and likely to be acceptable to app users. In contrast, the BCT of “anticipated regret” was viewed as potentially practicable to deliver within an app but unlikely to be acceptable to app users, who might view it as “guilt tripping” them about taking their medication, potentially leading to disengagement with the intervention.

### Ethical Considerations

This study did not involve human participants, primary data collection, observations of public behavior, or secondary analysis of previously collected individual-level data. All information analyzed was obtained from existing literature reviews and the published results of expert consensus exercises. The judgments and interpretations presented were made solely by the study authors as part of the analytic methodology and do not constitute human subjects research. As such, institutional review board approval and informed consent were not required. No individuals were recruited or compensated.

## Results

### Step 1: Identifying Influences on Medication Adherence

The review conducted by Asthma + Lung UK and Public Health England identified 31 influences on preventer inhaler adherence (Table 1).

**Table 1.** Identified influences on preventer inhaler adherence mapped to mechanisms of action.

Influence on adherence	Example	Mapping of influence to the mechanisms of action defined by Michie et al [24]
Limited awareness that there is a need to take inhalers even if symptoms are controlled	“I think if it’s quite severe, you have to control it, it’s a real part of your life in terms of medication, everything, lifestyle. Whereas if it’s mild, it rarely flares up, but when it does, then you really manage it to the limit.”	Knowledge
Confusion about how much to take and when	“But obviously it says two puffs on there, but how many is too many?”	Knowledge
Confusion over which inhaler to take when if multiple	“There are so many different pumps, by the time you’ve gone through every single colour, which one’s got steroids, which one hasn’t, you must be thinking ‘I’m going to die anyway.’”	Knowledge
Change environment to accommodate inhalers (eg, change of bag size)	“If you want to put a spacer as well as inhalers, etc, in it, you would need to go for a large one. I have a pretty toiletry bag. It keeps everything together and clean.”	Environmental context and resources
Formation of habits as a result of taking regularly	“Having a fixed daily routine can promote adherence”	Behavioral cuing
Always have inhaler to hand or deliberately having multiple inhalers in different places	“So, I’ve done functional things, like I’ll leave my asthma pump at my work drawer, places where you can go and leave things”	Environmental context and resources
Use of prompts and reminders	“Using environmental cues to trigger habitual taking of medication, such as reminder devices … can promote adherence”	Behavioral cuing
Symptoms serve as a reminder	“Experiencing symptoms serves as a reminder to use inhalers”	Behavioral cuing
Preference to use natural/herbal remedies over medication	“Patients may prefer to use folk remedies, behavioural strategies, and religious approaches before progressing to prescription medications”	Attitude toward the behavior
Viewing asthma outcomes as having more to do with faith/chance than treatment effectiveness	“Barriers…included the wrong beliefs [that]… asthma is more subject to faith and chance than to treatment effectiveness”	Beliefs about consequences
Forgetting to use them	“You just don’t even remember needing to take the pump”	Memory, attention, and decision processes
Interpretation of respiratory symptoms as normal variation, rather than due to asthma	“Even people in general sometimes have trouble breathing...I don’t know what’s normal”	Memory, attention, and decision processes [regarding interpreting symptoms] and knowledge [of what’s normal]
Using own judgment (over suggestions from professionals/medical tools)	“I might have had a low peak flow for a couple of days. But it kept instructing me to increase the dose, and I did not think it was necessary”	Memory, attention, and decision processes
Significant life event/asthma event prompts use	“Patients tend to be motivated to manage their asthma…when asthma affects a valued activity. Some were not motivated to act until it posed a life-threatening state”	MoA^a^ depends on how the person interprets the meaning of that event. For asthma events, the MoA seems likely to be perceived susceptibility/vulnerability.
Stigma/social judgment for using inhalers in public	“Asthma patients never feel comfortable to use asthma medications especially inhaler in public because by this act they feel stigmatized.”	Social influences
Opinions of friends/family/media (doubt from others that inhaler is needed	“Attitudes toward taking asthma medications are influenced by friends, family, the media”	Subjective norms
Poor health care professional/patient communication	“When I go to my appointment, they try to rush, get you in there, rush you out, get you some stuff, ‘Take this, do this.’”	Social influences
Lack of time to take inhalers (daily life)	“At least one squirt and it does something. Not take two, breathe in for 10, hold that. You haven’t got time. You really don’t.”	Environmental context and resources
Inhalers out of date due to irregular use	“Yes, I’ve had one of them but by the time I get round to taking it, they’re out of date, so…”	Environmental consequences and resources
Not wanting to identify as a ‘sick’ person	“I hate feeling like I’m different. I have a problem when it comes to that, and I hold off on my medicine.”	Social/professional role and identity, also self-image
Level of acceptance of asthma as part of identity	“It depends on whether you’ve accepted asthma as part of your identity”	Self-image
Not wanting to feel controlled by asthma/dependent on medication	“Restricting use of asthma medicines (to not feel controlled or dependent on medicines)”	Self-image, also beliefs about consequences if the person perceives that a consequence of adherence is feeling controlled/dependent
Doubt about asthma diagnosis	“In terms of her diagnosis, she doesn’t believe it addressed the actual condition she had, and she still believes it doesn’t”	General attitude/belief, self-image
Confidence in using inhalers	“Medication adherence for asthma is strongly influenced by self-efficacy levels”	Beliefs about capabilities
Perceived efficacy of inhalers	“Adherence is positively affected by the belief that giving inhaled corticosteroids would protect the patient from getting worse”	Beliefs about consequences
Beliefs about side effects	“At least 50% refused to adhere to their prescribed therapy fully because of concerns over side effects”	Beliefs about consequences
Concern that inhalers do more harm than good	“Barriers related to these facts included the wrong beliefs; for instance, asthma medicines may be more harmful than beneficial”	Beliefs about consequences
Perceiving cause and effect of using inhalers on asthma symptoms	“I’ve had times when I have been on strict medication regimes and I have still had attacks”	Beliefs about consequences
Embarrassment (eg, of using inhalers in public)	“They identified some particular issues such as …embarrassment over medication use in public“	Beliefs about consequences and emotions (embarrassment has both cognitive and emotional components)
Discomfort with long-term use of inhalers	“One factor in their lack of adherence was high discomfort with long-term use of ICS”	Beliefs about consequences and emotions (discomfort has both cognitive and emotional components)
Prioritizing asthma to support	“I’ve got 2 kids; I cannot afford to be sick, I cannot afford not to have the energy to work and to do all of these things. So, I will take it”	Goals

a MoA: mechanism of action.

### Step 2: Mapping Influences to Behavior Change Intervention Mechanisms of Action

Each of the influences identified above was then mapped to the definitions of the 26 MoAs used in the Theory and Techniques tool [22,24]. The mappings are shown in the third column of Table 1. Some influences were judged to contain elements of more than one MoA. For example, “Interpretation of respiratory symptoms as normal variation, rather than due to asthma” was judged to map to both memory, attention, and decision process (as interpreting one’s symptoms is a decision) and to knowledge (of what is normal variation in respiratory symptoms).

### Step 3: Mapping Mechanism of Actions to Behavior Change Techniques That Expert Consensus Suggests Will Change Each Mechanism of Action

Each MoA was linked to between 1 and 11 BCTs that might potentially work to change it (please see Table S3 in Multimedia Appendix 1 for details). In total, 41 BCTs were identified as possible candidates for inclusion in an app.

### Step 4: Filtering BCTs in Terms of Their Likely Acceptability and Practicability

The final set of BCTs judged to be acceptable and practicable to change each mechanism of action is shown in Table 2. There were between 0 and 5 BCTs judged practicable and acceptable to change a MoA when delivered as part of a preventer inhaler adherence app for people with asthma, depending on the MoA. The reasoning behind the inclusion or exclusion of each BCT identified in the previous step can be viewed in Table S3 in Multimedia Appendix 1.

**Table 2.** Final set of recommended behavior change techniques to change different influences on preventer inhaler use.

Influence on adherence	Closest mechanism of action	Final recommended BCTs^a^^,^^b^
Limited awareness that there is a need to take inhalers even if symptoms are controlled	Knowledge	5.1 information about health consequences
Confusion about how much to take and when	Knowledge	4.1 instruction on how to perform the behavior
Confusion over which inhaler to take when if multiple	Knowledge	4.1 instruction on how to perform the behavior 5.1 information about health consequences
Change environment to accommodate inhalers (eg, change of bag size)	Environmental context and resources	12.1 restructuring the physical environment
Formation of habits as a result of taking regularly	Behavioral cuing	1.4 action planning 7.1 prompts/cues 8.3 habit formation 12.1 restructuring the physical environment
Always have inhaler to hand/deliberately having multiple inhalers in different places	Environmental context and resources	7.1 prompts/cues (for having inhaler to hand) 12.1 restructuring the physical environment (for having multiple inhalers in useful locations)
Use of prompts and reminders	Behavioral cuing	1.4 action planning 7.1 prompts/cues 8.3 habit formation 12.1 restructuring the physical environment
Symptoms serve as a reminder	Behavioral cuing	1.4 action planning 7.1 prompts/cues 8.3 habit formation
Preference to use natural/herbal remedies over medication	Attitude toward the behavior	5.1 information about health consequences 9.1 credible source 9.2 pros and cons (when accompanied by at least one of the two other BCTs listed in this column)
Viewing asthma outcomes as having more to do with faith/chance than treatment effectiveness	Beliefs about consequences	5.1 information about health consequences 5.2 salience of consequences 5.3 information about social and environmental consequences 5.6 provide information about emotional consequences 9.2 pros and cons
Forgetting to use them	Memory, attention, and decision processes	7.1 prompts/cues
Interpretation of respiratory symptoms as normal variation, rather than due to asthma	Memory, attention, and decision processes (for deciding on the meaning of symptoms) Knowledge (of what is normal variation)	Memory, attention, and decision processes N/A^c^ Knowledge 5.1 information about health consequences
Using own judgment (over suggestions from professionals/medical tools)	Memory, attention, and decision processes	N/A
Significant life event/asthma event prompts use	Depends how the person interprets the meaning of that event. Possibly perceived susceptibility/vulnerability.	5.2 salience of consequences
Stigma/social judgment for using inhalers in public	Social influences	3.1 social support (unspecified) 3.2 social support (practical) 6.3 information about others’ approval
Opinions of friends/family/media (doubt from others that inhaler is needed)	Subjective norms	6.3 information about others’ approval
Poor health care provider/patient communication	Social influences	3.1 social support (unspecified) 3.2 social support (practical) 6.3 information about others’ approval
Lack of time to take inhalers (daily life)	Environmental context and resources	3.2 social support (practical) 12.1 restructuring the physical environment
Inhalers out of date due to irregular use	Environmental context and resources	N/A, suggest tackling this barrier by enhancing adherence by other means so that inhalers do not go out of date
Not wanting to identify as a “sick” person	Social/professional role and identity, possibly also self-image	3.1 social support (unspecified) 6.2 social comparison 9.1 credible source
Level of acceptance of asthma as part of identity	Self-image	13.1 identification of self as a role model
Not wanting to feel controlled by asthma/dependent on medication	Possible belief about consequences if a consequence of adherence is feeling controlled/dependent, self-image	Beliefs about consequences: 5.1 information about health consequences 5.2 salience of consequences 5.3 information about social and environmental consequences 5.6 information about emotional consequences 9.2 pros and cons Self-image: N/A
Doubt about asthma diagnosis	General attitude/belief and self-image	General attitude/belief: 9.1 credible source 9.2 pros and cons Self-image: N/A
Confidence in using inhalers	Beliefs about capabilities	4.1 instruction on how to perform the behavior 6.1 demonstration of the behaviour 8.1 behavioural practice/rehearsal 15.1 verbal persuasion about capability 15.3 focus on past success
Perceived efficacy of inhalers	Beliefs about consequences	5.1 information about health consequences 5.2 salience of consequences 5.3 information about social and environmental consequences 5.6 information about emotional consequences
Beliefs about side effects	Beliefs about consequences	5.1 information about health consequences 5.2 salience of consequences (possibly) 9.2 pros and cons
Concern that inhalers do more harm than good	Beliefs about consequences	5.1 information about health consequences 5.2 salience of consequences 9.2 pros and cons
Perceiving cause and effect of using inhalers on asthma symptoms	Beliefs about consequences	5.1 information about health consequences 5.2 salience of consequences
Embarrassment (eg, of using inhalers in public)	Beliefs about consequences Emotions	Beliefs about consequences: 5.2 salience of consequences 5.3 information about social and environmental consequences 5.6 information about emotional consequences 9.2 pros and cons Emotions 11.2 advise on ways to reduce negative emotions to facilitate performance of the behavior
Discomfort with long-term use of inhalers	Beliefs about consequences and emotions	5.1 information about health consequences 5.2 salience of consequences 5.6 information about emotional consequences 9.2 pros and cons 11.2 reduce negative emotions
Prioritizing asthma to support	Goals	1.3 goal setting (outcome)

a Behavior change techniques are numbered using the numbering system from the BCT Taxonomy v1.

b BCT: behavior change technique.

c N/A: not applicable.

## Discussion

### Principal Findings

We have used a systematic approach, grounded in behavioral science, to create a shortlist of BCTs that app developers can use to target particular influences on preventer inhaler adherence in people with asthma. We identified 24 BCTs that might be effective, practical, and acceptable to deliver via an asthma medication adherence app to target specific influences on adherence. In contrast, 1 systematic review of existing adherence apps for patients with asthma identified only 9 BCTs in the included apps [16] while the review of 23 asthma apps by Ramsey et al [17] found a mean number of 4 BCTs per app. However, these BCTs were not linked to specific influences on nonadherence in any logical way. In similar ways, a more recent review of asthma mHealth apps [18] did not consider how appropriate the included BCTs were for altering the influences on asthma self-management targeted by the apps. In contrast, the behavioral influence-targeting approach used in this study can be used more broadly in designing apps and other mHealth interventions targeting both medication adherence and other forms of behavior change, ensuring intervention components are appropriate for the factors influencing the relevant behavior.

There are several strengths to our approach. First, we started with a comprehensive set of the influences on preventer inhaler use based on previous research. Second, we used a tool incorporating the findings of a large expert consensus study to identify BCTs potentially effective at changing specific influences. This increases the chances that an app will be effective at changing the influences on adherence behavior. Our approach, nevertheless, has some limitations. While the set of influences was based on a synthesis of previous research findings, there are a number of limitations to that synthesis. First, time and resource considerations led to the use of rapid review methods and meant that many of the analytic decisions involved only 2 researchers. Second, much of the available evidence came from qualitative studies, which meant it was not possible to identify which of the 31 influences might have the greatest impact on adherence. Third, the only gray literature included was that readily available to Asthma UK. A more extensive search for unpublished literature could have augmented the synthesis results. Finally, the strongest evidence for the impact of a potential influence on adherence comes from an experimental study which shows that changing the levels of the influence (eg, reducing concerns about side effects) alters subsequent adherence. None of the studies included in the research synthesis had this design. Future research in this area should concentrate on generating such evidence.

Ideally, the choice of a BCT to change a particular MoA would have been based on evidence showing that the BCT changed the MoA, which in turn enhanced medication adherence. However, such evidence does not yet exist for every BCT-MoA-adherence combination that app designers might be interested in, hence the present need to rely on expert consensus. Future app evaluation studies can contribute to this evidence base by using experimental designs and assessing changes in the relevant MoAs as well as adherence behavior. Behavioral scientists will need to pursue additional work to develop reliable and valid measures of key MoAs that can be used in such evaluation studies.

We assumed that the app needed to be easy to use with very little input from health care professionals. We may have reached different conclusions about which BCTs were practicable if we had anticipated that the app was going to be used as one component of an adherence intervention that also involved dedicated sessions with a health care provider. The judgments of the potential practicability and acceptability of the different BCTs will always involve a subjective element [25,26]. However, they were based on the team’s experience with behavior change interventions to promote medication adherence and digital technologies. Nevertheless, this approach could have been strengthened further by including the views of people with lived experience of asthma and adherence difficulties.

For app developers, there are some recommendations that arise from our findings. First, since the reasons for nonadherence differ across individuals, we would recommend that an app should start by using a relatively simple assessment of the individual user’s reasons for their low inhaler use. This could be in the form of a simple questionnaire [27] or a list of common reasons (eg, forgetting, low belief in need for daily inhaler use, concerns regarding steroids). This could then be linked to an algorithm which would indicate which BCT would be best to target that reason, as per Table 2. For example, if the user indicates that they are not aware that they need to use their preventer inhaler every day (influence 1 in Table 2), then the recommended BCT would be for the app to provide information incorporating the likely health consequences, such as worsening symptoms or possible hospital emergency visits. Alternatively, if the initial screening reveals that the user does not have a regular habit or pattern of daily inhaler use (row 6 of Table 2), then an “action planning” BCT is indicated as an app component. This would require the user to specify a consistent time and place when the inhaler will be used, linked to an existing daily habit such as toothbrushing. If a user indicates a number of reasons for their nonadherence, then the app will need to ask them to prioritize these in order of importance so that the targeted BCTs can be determined and delivered accordingly. For the more practical issue of how to operationalize the BCT within the app, the final column of table S3 provides details of what each BCT entails, as per its definition in the BCT Taxonomy [7]. App developers should clearly state which BCTs their app includes to tackle which influences in its documentation, to allow apps to be appropriately classified in any future repositories of medication adherence technologies [28]. Health care decision-makers concerned with whether to endorse particular asthma adherence apps for patient use in a given health care system can also make use of our findings. For example, they might appraise the extent to which each candidate app tackles the range of influences on adherence, and whether the app applies appropriate BCTs to alter those influences, as per Table 2.

In addition to selecting the most appropriate BCT for targeting a particular cause of nonadherence, apps need to take account of the phase of an individual’s inhaler use from early initiation to implementation (regular use as part of a daily routine) and persistence (continued inhaler use even in the absence of symptoms) [29]. Since different influences may be more important at each phase, this could be a part of the initial assessment component of the app. For example, if the app user indicates an initial antipathy toward using an inhaler possibly combined with a preference for “natural” remedies (influence 9 in Table 2), this is a likely cause of noninitiation of treatment, and so the recommended BCTs would be a combination of information about the health consequences of not starting treatment provided by a credible source (eg, expert patient and/or doctor/pharmacist). However, if the individual makes a good start with their inhaler but then decides to stop regular use due to both feeling better and concerns about possible harms from the inhaler (row 29 in Table 2), then the most relevant BCTs would involve a combination of information about the salient consequences of stopping treatment together with ways of enabling them to weigh up the pros and cons of regular inhaler use.

While we have identified a set of BCTs that may be useful to include, many creative decisions remain for app designers about how to implement each BCT. The impact of BCTs on medication adherence is likely to depend on their style of delivery, ie, the manner in which intervention content is communicated [30,31]. Because people are motivated by their needs to feel competent, autonomous, and related to others [32], intervention content that is delivered in a nonjudgmental, collaborative fashion that supports patient autonomy may lead to greater behavior change than the same intervention content delivered in a moralizing, didactic, or controlling style. App designers will also want to consider incorporating features that promote app engagement [33]. Greater daily engagement with a medication adherence app was found to predict greater objectively measured medication adherence in people with asthma and chronic obstructive pulmonary disease in 1 study [34]. Obtaining feedback from people with lived experience of asthma and adherence difficulties will be essential to ensure that the implementation of BCTs within a prototype app is acceptable to and engaging for potential users.

### Conclusions

This paper has used a systematic process to identify the optimal BCTs to tackle specific influences on preventer inhaler nonadherence via an app-based intervention. Its findings are useful for app designers and others interested in digital health interventions to promote medication adherence.

## Supplementary material

10.2196/49348 Multimedia Appendix 1 Supplementary material including list of selected behavior change techniques.

## Abbreviations

APEASE: Acceptability, Practicability, Effectiveness and cost-effectiveness, Affordability, Side effects/safety, and Equity
BCT: behavior change technique
MoA: mechanism of action
