# Supplementary material for: Selection of Behavior Change Techniques for Asthma Medication Adherence Apps: Evidence-Based Design Study
Source: JMIR Mhealth Uhealth. 2025 Sep 23;13:e49348. doi: 10.2196/49348 (PMC12456459; doi:10.2196/49348)
Supplement: Multimedia Appendix 1 [file mhealth-v13-e49348-s001.docx]

**Multimedia appendix**

**Table S1: Unpublished behavioural insights research commissioned by Asthma UK that informed the identification of barriers and enablers to preventer inhaler adherence (“gray literature”).**

| **Document details** | **Description** |
| --- | --- |
| Title: Living with Severe Asthma Final Full with appendix.pdf  Supplemental: Severe asthma – Empathy & Experience maps  Date: 2019 | This document describes the results of depth interviews with people with severe asthma. It describes detailed findings, including those around symptom management practices, the evaluative stances that people adopt in relation to their asthma and other factors, relationships with medications, and social factors. |
| Title: Asthma Workshops - Final Report  Date: 2019 | Asthma UK commissioned further insight work to explore groups who were less actively engaged with their asthma. |
| Title: Arete Report Final.pdf  Supplemental: 2018-09 Arete focus group - Transcription Final.pdf  Date: 2018 | Arete Medical Technologies commissioned Asthma UK to facilitate insight via a series of focus groups. The aim of the research included exploring self-management and monitoring approaches by gathering insight on self-management and monitoring behaviour, including challenges and barriers, processes and routines and the tools used. |
| Title: Engaging the Unengaged.pdf  Date: 2017  Supplemental: Engaging the unengaged poster European Respiratory Society 2018.pdf  Date: 2018 | Asthma UK commissioned some insight work to explore how they might work with people who are less actively engaged with their asthma, and with Asthma UK. |
| Title: Asthma Attack Reduction Programme Focus Groups Report.pdf  Date: 2016 | This document relates to the Asthma Attack Reduction Programme (AARP): a behaviour change programme to improve asthma self-management and adherence to asthma medicines for adults of working age at a higher risk of an asthma attack. Focus groups were conducted to address gaps in the evidence base, including specific beliefs about the value/necessity of asthma medicines that were relevant to reducing non-adherence. |
| Title: Social Listening Asthma Reviews - appendices.docx  Date: 2015 | This document presents an analysis of discussions from patient.co.uk and asthma.org.uk online patient forums. |

**Additional information on the methods used for the rapid review**

**Search strategy**

The search strategy was developed by the Public Health England (PHE) Behavioural Insights Team in consultation with the Asthma UK and the PHE Knowledge and Library Services (KLS). The search strategy was designed to identify literature pertaining to the target behaviour.

Four databases (EMBASE, Medline, PsychINFO and Psychology & Behavioral Sciences Collection) were searched between January 1996 (as per an earlier published review by Miles et al, 2017) and 5 August 2020. The search strategies were adapted for the individual databases searched. The search strategy comprised of search terms that covered the following concepts and their synonyms: ‘asthma’, ‘self-care’ and ‘self-management’, ‘barriers’ and ‘facilitators’, and was supplemented by additional searches using terms related to ‘medication adherence’ and ‘inhaler’. For details of the search terms used for each database, please see Supplementary Table 2. The search was finalised and conducted by the PHE KLS Team.

**Study selection process**

De-duplicated search results (titles, abstracts, and meta-data) were entered into Endnote for initial screening involving identification of reviews (search conducted on title or abstract for the words “review” and “meta-analysis”). The resulting title and abstracts were screened, with conference proceedings and abstracts removed.

**Inclusion criteria**

To be included in the rapid review, the reviews had to report findings related to:

1. Adults with asthma (the full-text had to clearly point to a large proportion of adults living with asthma included in the review, and/or offer the possibility to identify data relevant to the possible to identify factors relevant to adults)
2. Patients’ perspectives (not healthcare professionals’ or carers)
3. A review or meta-analyses of any study design (i.e. qualitative, survey, intervention)
4. Modifiable influences (barriers/facilitators) or associations that correlate/predict engagement with the target behaviours
5. Full text in English language

**Exclusion criteria**

1. Non-asthma populations or asthma a minority or impossible to determine which data relates to persons living to asthma
2. Target population: healthcare professionals, children, adolescents, carers
3. Reporting only effectiveness and safety of interventions
4. Reporting only intervention development or content
5. Conference proceedings, abstracts and theses

Two reviewers independently reviewed abstracts and classified the reviews as ‘include’, ‘exclude’ and ‘maybe’. Reviews for which there was an agreement to exclude were excluded. The full-text of the remaining reviews was obtained. Full text review was conducted by one reviewer in consultation with a second reviewer.

**Table S2: Search terms used in the rapid review for each database searched.**

| **Database** | **Embase** | **Medline** | **PsycInfo** | **Psychology & Behavioral Sciences Collection** |
| --- | --- | --- | --- | --- |
| **Indicator** | exp *asthma/ | exp *Asthma/ | exp *Asthma/ | DE "ASTHMA" |
|  | asthma* | asthma* | asthma* |  |
|  |  |  | exp *Treatment Barriers/ |  |
| **Barriers** | (barrier* or facilitator*) | (barrier* or facilitator*) | (barrier* or facilitator* or influenc* or predictor* or enabler or association) | (barrier* or facilitator* or influenc* or predictor* or enabler or association) |
| **Attitudes** | exp *patient decision making/ | exp *choice behavior/ | exp *choice behavior/ | (DE "CHOICE (Psychology)")  OR (DE "BELIEF change") |
|  | *attitude to health/ | exp *attitude to health/ | *client attitudes/ | DE "PATIENTS' attitudes" |
|  | (attitude* or choice* or decision*) | (attitude* or choice* or decision*) | (attitude* or choice* or decision*) |  |
| **Behaviours** |  |  |  |  |
| ***Medication*** | exp *medication compliance/ | Medication Adherence/ | treatment compliance/ | DE "PATIENT compliance" |
|  | ((uptake or tak* or adher* or initiat* or us$3 or comply or compliance) adj2 inhaler*) | ((uptake or tak* or adher* or initiat* or us$3 or comply or compliance) adj2 inhaler*) | ((uptake or tak* or adher* or initiat* or us$3 or comply or compliance) adj2 inhaler*) |  |
| ***Self care*** | exp *self care/ | exp *Self Care/ | self management/ | DE "SELF-efficacy" |
|  | ("self assessment" or "self management" or "self efficacy"  or "self care" or "self monitor*") | ("self assessment" or "self management" or "self efficacy" or "self care" or "self monitor*") | exp *Self-Efficacy/ | DE "SELF-monitoring (Psychology)" |
|  | ((monitor* or measur* or us$3 or tak*) adj2 "peak flow") | ((monitor* or measur* or us$3 or tak*) adj2 "peak flow") | ("self assessment" or "self management" or "self efficacy" or "self care" or "self monitor*") | ((monitor* or measur* or use or using or tak*) N2 "peak flow") |
|  | (us$3 adj1 "assessment tool*") | (us$3 adj1 "assessment tool*") | ((monitor* or measur* or us$3 or tak*) adj2 "peak flow") | ((use or using) N1  "assessment tool*") |
|  | (behavio?r adj2 regulat*) | (behavio?r adj2 regulat*) | (us$3 adj1 "assessment tool*") | (behavio?r N2 regulat*) |
|  |  |  | (behavio?r adj2 regulat*) |  |
| **Limits** |  |  |  |  |
|  | 1996-2020 | 1996-2020 | 1996-2020 | 1996-2000 |
|  | human | human |  |  |

**Reviews included in the rapid review**

Ahmad, S., & Ismail, N. E. (2015). Stigma in the lives of asthma patients: a review from the literature. *International Journal of Pharmacy and Pharmaceutical Sciences*, *7*(7), 40-6.

Ahmed, S., Steed, L., Harris, K., Taylor, S. J., & Pinnock, H. (2018). Interventions to enhance the adoption of asthma self-management behaviour in the South Asian and African American population: a systematic review. *NPJ primary care respiratory medicine*, *28*(1), 5.

Alicea-Alvarez, N., Swanson-Biearman, B., & Kelsen, S. G. (2014). A review of barriers to effective asthma management in Puerto Ricans: cultural, healthcare system and pharmacogenomic issues. *Journal of Asthma*, *51*(1), 97-105.

Amin, S., Soliman, M., McIvor, A., Cave, A., & Cabrera, C. (2020). Understanding patient perspectives on medication adherence in asthma: a targeted review of qualitative studies. *Patient preference and adherence*, 541-551.

Andrews, K. L., Jones, S. C., & Mullan, J. (2014). Asthma self management in adults: a review of current literature. *Collegian*, *21*(1), 33-41.

Dima, A. L., Hernandez, G., Cunillera, O., Ferrer, M., & de Bruin, M. (2015). Asthma inhaler adherence determinants in adults: systematic review of observational data. *European Respiratory Journal*, *45*(4), 994-1018.

Holgate, S. T., Price, D., & Valovirta, E. (2006). Asthma out of control? A structured review of recent patient surveys. *BMC pulmonary medicine*, *6*, 1-9.

Miles, C., Arden-Close, E., Thomas, M., Bruton, A., Yardley, L., Hankins, M., & Kirby, S. E. (2017). Barriers and facilitators of effective self-management in asthma: systematic review and thematic synthesis of patient and healthcare professional views. *NPJ primary care respiratory medicine*, *27*(1), 57.

Morton, K., Dennison, L., May, C., Murray, E., Little, P., McManus, R. J., & Yardley, L. (2017). Using digital interventions for self-management of chronic physical health conditions: a meta-ethnography review of published studies. *Patient education and counseling*, *100*(4), 616-635.

Yii, A. C., & Koh, M. S. (2013). A review of psychological dysfunction in asthma: affective, behavioral and cognitive factors. *Journal of Asthma*, *50*(9), 915-921.

**Table S3: Identification of practicable and acceptable behaviour change techniques to target influences on preventer inhaler adherence.**

Assumptions re context for an app-based intervention to inform practicability judgments:

(1) The app should work as a standalone intervention. Although it might be recommended to a patient by a HCP, it will not require further HCP involvement;

(2) The app should not require the user to have any further technology beyond their smartphone (e.g. a laptop or a way of monitoring lung functioning);

(3) The app is to be rolled out in a high-income country where asthma is largely managed in primary care (e.g. the UK)

| **Influence on adherence** | **example** | **Nearest MoA from the Theories & Techniques Tool** | **BCTs suggested by the Theories and Techniques Tool^a^** | **Practicability of BCT delivery via an app** | **Acceptability of BCT to target population^b^** | **Final recommended BCTs** | **Definitions of recommended BCTs (from the BCT Taxonomy v1, Michie et al, 2013)** |
| --- | --- | --- | --- | --- | --- | --- | --- |
| **Limited awareness that there is a need to take inhalers even if symptoms are controlled** | *“I think if it’s quite severe, you have to control it, it’s a real part of your life in terms of medication, everything, lifestyle. Whereas if it’s mild, it rarely flares up, but when it does, then you really manage it to the limit”* | Knowledge | - 2.6 biofeedback - 4.1 instruction on how to perform the behaviour - 4.2 information about antecedents - 5.1 information about health consequences - 5.3 information about social and environmental consequences | - 2.6 - would need some kind of lung function measure via the app e.g. an app using phone microphone – this technology is still in early stages and under in development, therefore currently judged not practicable - 4.1 - not relevant to this influence - 4.2 not relevant to this influence - 5.1 practicable - 5.3 less relevant to this influence | - 5.1 acceptable, as long as avoid overly didactic style | - **5.1 information about health consequences** | - 5.1 Provide information (e.g. written, verbal, visual) about health consequences of performing the behaviour |
| **Confusion about how much to take and when** | *“But obviously it says two puffs on there, but how many is too many?”* | Knowledge | - 2.6 biofeedback - 4.1 instruction on how to perform the behaviour - 4.2 information about antecedents - 5.1 information about health consequences - 5.3 information about social and environmental consequences | - 2.6 not relevant to this influence - 4.1 would need to tailor to specific regime - 4.2 not relevant to confusion - 5.1 not relevant, - 5.3 not relevant to this type of knowledge | - 4.1 would be acceptable if tailored | - **4.1 instruction on how to perform the behaviour** | - 4.1 Advise or agree on how to perform the behaviour |
| **Confusion over which inhaler to take when if multiple** | *“There are so many different pumps, by the time you’ve gone through every single colour, which one’s got steroids, which one hasn’t, you must be thinking I’m going to die anyway.”* | Knowledge | - 2.6 biofeedback - 4.1 instruction on how to perform the behaviour - 4.2 information about antecedents - 5.1 information about health consequences - 5.3 information about social and environmental consequences | - 2.6 not relevant to this influence - 4.1 would need to tailor to specific regime - 4.2 not relevant to confusion - 5.1 feasible to provide information about which inhaler tackles what health outcome (prevention vs. symptom relief), - 5.3 not relevant to this type of knowledge | - 4.1 acceptable if tailored - 5.1 acceptable | - **4.1 instruction on how to perform the behaviour** - **5.1 information about health consequences** | - 4.1 Advise or agree on how to perform the behaviour - 5.1 Provide information (e.g. written, verbal, visual) about health consequences of performing the behaviour |
| **Change environment to accommodate inhalers (e.g. change of bag size)** | *“If you want to put a spacer as well as inhalers etc in it you would need to go for a large one. I have a pretty toiletry bag. It keeps everything together and clean”* | Environmental context and resources | - 3.2 social support (practical) - 7.1 prompts/cues - 7.5 remove aversive stimulus - 12.1 restructuring the physical environment - 12.2 restructuring the social environment - 12.3 avoiding/reducing exposure to cues for the behaviour - 12.5 adding objects to the environment | - 3.2 not relevant - 7.1 not relevant - 7.5 not relevant, - 12.1 - can either be an actual restructuring of the environment OR advising on changing the physical environment. The latter might be more feasible. - 12.2 not relevant - 12.3 not relevant - 12.5 requires physically adding an object - hard to implement this digitally for this MoA | - 12.1 acceptable | - **12.1 restructuring the physical environment** | - 12.1 Change, or advise to change the physical environment in order to facilitate performance of the wanted behaviour or create barriers to the unwanted behaviour (other than prompts/cues, rewards and punishments) |
| **Formation of habits as a result of taking regularly** | *“Having a fixed daily routine, can promote adherence”* | Behavioural cueing | - 1.4 action planning - 7.1 prompts/cues - 8.3 habit formation - 12.1 restructuring the physical environment - 12.3 avoidance/reducing exposure to cues for the behaviour - 12.5 adding objects to the environment | - 1.4 possible, useful for helping people work out what times/places they will take their preventer inhaler - 7.1 feasible - use digital prompts - 8.3 feasible - 12.1 - advising on changes possible - 12.3 not relevant - 12.5 less relevant to this particular influence | - 1.4 acceptable, - 7.1 acceptable, - 8.3 acceptable, - 12.1 acceptable | - **1.4 action planning** - **7.1 prompts/cues** - **8.3 habit formation** - **12.1 restructuring the physical environment** | - 1.4 Prompt detailed planning of performance of the behaviour (must include at least one of context, frequency, duration and intensity). Context may be environmental (physical or social) or internal (physical, emotional or cognitive) - 7.1 Introduce or define environmental or social stimulus with the purpose of prompting or cueing the behaviour. The prompt or cue would normally occur at the time or place of performance - 8.3 Prompt rehearsal and repetition of the behaviour in the same context repeatedly so that the context elicits the behaviour 12.1 Change, or advise to change the physical environment in order to facilitate performance of the wanted behaviour or create barriers to the unwanted behaviour (other than prompts/cues, rewards and punishments) |
| **Always have inhaler to hand/ deliberately having multiple inhalers in different places** | *“So, I've done functional things, like I'll leave my asthma pump at my work drawer, places where you can go and leave things”* | Environmental context and resources | - 3.2 social support (practical) - 7.1 prompts/cues - 7.5 remove aversive stimulus - 12.1 restructuring the physical environment - 12.2 restructuring the social environment - 12.3 avoiding/reducing exposure to cues for the behaviour - 12.5 adding objects to the environment | - 3.2 not relevant - 7.1 could tie something person always does when leaving the house with making sure they have their inhaler or could add new prompt (e.g. app reminder) - 7.5 not relevant, - 12.1 - can either be an actual restructuring of the environment OR advising on changing the physical environment. The latter might be more feasible. People may need suggestions/support to identify best place to leave extra inhaler. - 12.2 not relevant - 12.3 not relevant - 12.5 requires physically adding an object having identified a suitable place in the environment so can’t be done via app | - 7.1 acceptable, - 12.1: for having multiple inhalers in different places need to consider ease/cost of obtaining multiple inhalers may be a barrier for some. | - **7.1 prompts/cues (for having inhaler to hand)** - **12.1 restructuring the physical environment (for having multiple inhalers in useful locations)** | - 7.1 Introduce or define environmental or social stimulus with the purpose of prompting or cueing the behaviour. The prompt or cue would normally occur at the time or place of performance - 12.1 Change, or advise to change the physical environment in order to facilitate performance of the wanted behaviour or create barriers to the unwanted behaviour (other than prompts/cues, rewards and punishments) |
| **Use of prompts and reminders** | *“Using environmental cues to trigger habitual taking of medication, such as reminder devices … can promote adherence”* | Behavioural cueing | - 1.4 action planning - 7.1 prompts/cues - 8.3 habit formation - 12.1 restructuring the physical environment - 12.3 avoidance/reducing exposure to cues for the behaviour - 12.5 adding objects to the environment | - 1.4 doable, useful for helping people work out what prompts they want to have at what times - 7.1 feasible - use digital prompts - 8.3 feasible with repeated digital prompts - 12.1 - advising on changes more doable - 12.3 not relevant - 12.5 less relevant | - 1.4 acceptable, - 7.1 acceptable, - 8.3 acceptable - 12.1 acceptable | - **1.4 action planning** - **7.1 prompts/cues** - **8.3 habit formation** - **12.1 restructuring the physical environment** | - 1.4 Prompt detailed planning of performance of the behaviour (must include at least one of context, frequency, duration and intensity). Context may be environmental (physical or social) or internal (physical, emotional or cognitive) - 7.1 Introduce or define environmental or social stimulus with the purpose of prompting or cueing the behaviour. The prompt or cue would normally occur at the time or place of performance - 8.3 Prompt rehearsal and repetition of the behaviour in the same context repeatedly so that the context elicits the behaviour - 12.1 Change, or advise to change the physical environment in order to facilitate performance of the wanted behaviour or create barriers to the unwanted behaviour (other than prompts/cues, rewards and punishments) |
| **Symptoms serve as a reminder** | *“Experiencing symptoms serves as a reminder to use inhalers”* | Behavioural cueing | - 1.4 action planning - 7.1 prompts/cues - 8.3 habit formation - 12.1 restructuring the physical environment - 12.3 avoidance/reducing exposure to cues for the behaviour - 12.5 adding objects to the environment | - 1.4 could use to help people identify times/places other than when experience symptoms to take their inhaler - 7.1 relevant - 8.3 relevant – need to form an alternative habit rather than inhaler use being cued by symptoms - 12.1 not relevant to this influence - 12.3 not relevant, - 12.5 less relevant for this determinant | - 1.4 acceptable if rationale clearly explained - 7.1 acceptable, - 8.3 acceptable | - **1.4 action planning** - **7.1 prompts/cues** - **8.3 habit formation** | - 1.4 Prompt detailed planning of performance of the behaviour (must include at least one of context, frequency, duration and intensity). Context may be environmental (physical or social) or internal (physical, emotional or cognitive) - 7.1 Introduce or define environmental or social stimulus with the purpose of prompting or cueing the behaviour. The prompt or cue would normally occur at the time or place of performance - 8.3 Prompt rehearsal and repetition of the behaviour in the same context repeatedly so that the context elicits the behaviour |
| **Preference to use natural/herbal remedies over medication** | *“Patients may prefer to use folk remedies, behavioral strategies and religious approaches before progressing to prescription medications”* | Attitude towards the behaviour | - 5.1 information about health consequences, - 5.3 information about social and environmental consequences - 9.1 credible source - 9.2 pros and cons - 13.2 framing/reframing | - 5.1 easily done, but getting message right would be key - 5.3 environmental consequences might be motivating for this group but hard to generate so probably not practicable - 9.1 could work well if could identify source perceived as credible by person with these beliefs but likely to support inhaler use (e.g. media doctor known for interest in holistic medicine) - 9.2 could offer opportunity to consider pros/cons of natural remedies over medication - 13.2 would be hard to do for this belief – could be unwieldy in an app and, in general, this is a technique that needs human input to deliver well | - 5.1 – acceptable as long as get the message right, not overly didactic - 9.1 acceptability depends on using appropriate credible source - 9.2 could be perceived as empowering but app developers may wish to first present some information from a credible source and then use this BCT. - 13.2 may l alienate intervention users with strongly held beliefs in natural remedies | - **5.1 information about health consequences** - **9.1 credible source** - **9.2 pros and cons (when accompanied by at least one of the two other BCTs listed in this column)** | - 5.1 Provide information (e.g. written, verbal, visual) about health consequences of performing the behaviour - 9.1 Present verbal or visual communication from a credible source in favour of or against the behaviour - 9.2 Advise the person to identify and compare reasons for wanting (pros) and not wanting to (cons) change the behaviour |
| **Viewing asthma outcomes as having more to do with faith/chance than treatment effectiveness** | *“Barriers…included the wrong beliefs; … asthma is more subject to faith and chance than to treatment effectiveness”* | Beliefs about consequences | - 5.1 information about health consequences - 5.2 salience of consequences - 5.3 information about social and environmental consequences - 5.5 anticipated regret - 5.6 information about emotional consequences - 9.2 pros and cons - 9.3 comparative imagining of future outcomes - 10.1 material incentive (behaviour) - 10.8 incentive (outcome) - 10.10 reward (outcome) | - 5.1 - easy to provide information about consequences of taking a chance with one's asthma - 5.2 – could increase salience via including a patient story in the app - 5.3 - also feasible to generate information about adverse social consequences - 5.5 could potentially be done by sharing a patient story of someone who regretted relying on chance/faith - 5.6 could also be feasible via sharing a narrative or asking person to reflect - 9.2 could be done as some sort of voting tool/list making tool in an app - 9.3 could include some type of guided imagery exercise instructions in the app – focused on potential future outcomes – but could accidentally reinforce relying on faith/chance – not practicable to delivery via app - 10.1 not relevant for this influence - 10.8 not relevant for this influence - 10.10 not relevant for this influence | - 5.1 acceptable as long as information well-balanced and human, not overly didactic - 5.2 could be acceptable as long as patient story is subtle, not excessively stressing the point - 5.3 acceptable as long as information well-balanced and human, not overly didactic - 5.5 anticipated regret induction might be seen as a “guilt trip” therefore low acceptability - 5.6 potentially acceptable, as long as presented subtly - 9.2 acceptable if clear rationale provided | - **5.1 information about health consequences** - **5.2 salience of consequences** - **5.3 information about social and environmental consequences** - **5.6 provide information about emotional consequences** - **9.2 pros and cons** | - 5.1 Provide information (e.g. written, verbal, visual) about health consequences of performing the behaviour - 5.3 Provide information (e.g. written, verbal, visual) about social and environmental consequences of performing the behaviour - 5.6 Provide information (e.g. written, verbal, visual) about emotional consequences of performing the behaviour - 9.2 Advise the person to identify and compare reasons for wanting (pros) and not wanting to (cons) change the behaviour |
| **Forgetting to use them** | *“You just don't even remember needing to take the pump”* | Memory, attention and decision processes | - 7.1 prompts/cues - 11.3 conserving mental resources | - 7.1 high - both in terms of helping people set up prompts and cues and the digital provision of prompts and cues - 11.3 less relevant to this influence | - 7.1 acceptable | - **7.1 prompts/cues** | - 7.1 Introduce or define environmental or social stimulus with the purpose of prompting or cueing the behaviour. The prompt or cue would normally occur at the time or place of performance |
| **Interpretation of respiratory symptoms as normal variation, rather than due to asthma** | *“Even people in general sometimes have trouble breathing . . . I don’t know what’s normal”* | Memory, attention and decision processes (for deciding on meaning of symptoms)  Knowledge (of what’s normal variation) | **Memory, attention & decision processes**   - 7.1 prompts/cues - 11.3 conserving mental resources   **Knowledge**   - 2.6 biofeedback - 4.1 instruction on how to perform the behaviour - 4.2 information about antecedents - 5.1 information about health consequences - 5.3 information about social and environmental consequences | **Memory, attention & decision processes**   - 7.1 less relevant to this influence - 11.3 less relevant   **Knowledge:**  2.6 not practicable to deliver via an app with presently available technology  4.1 not relevant to this influence  4.2 not relevant to this influence  5.1 practicable if phrased appropriately - health consequences of leaving symptoms untreated  5.3 less relevant for this influence | **Memory, attention & decision processes**   - N/A   **Knowledge**   - 5.1 acceptable if phrased appropriately | **Memory attention & decision processes**   - **N/A**   **Knowledge**   - **5.1 information about health consequences** | - 5.1 Provide information (e.g. written, verbal, visual) about health consequences of performing the behaviour |
| **Using own judgement (over suggestions from professionals/ medical tools)** | *“I might have had a low peak flow for a couple of days. But it kept instructing me to increase the dose, and I did not think it was necessary”* | Memory, attention and decision processes | - 7.1 prompts/cues - 11.3 conserving mental resources | - 7.1 practicable if have some means for people to record their daily symptoms so that this record could trigger an alert - 11.3 less relevant | - 7.1 likely to have low acceptability – someone who prefers to use their own judgement rather than medical tools may be hard to engage with any adherence intervention, so unlikely to be willing to record daily symptoms | **N/A** |  |
| **Significant life event/ asthma event prompts use** | *“Patients tend to be motivated to manage their asthma… when asthma affects a valued activity. Some were not motivated to act until it posed a life threatening state”* | Depends how person interprets the meaning of that event. Possibly perceived susceptibility/ vulnerability | - 5.1 information about health consequences - 5.2 salience of consequences | - 5.1 not relevant - person is already aware of consequences, doesn't need information about them - 5.2 easier to influence salience of these consequences - | - 5.2 potentially acceptable but will not be applicable to everyone. Needs handling with care - potential for traumatic memories being evoked without having means to support people with them. | - **5.2 salience of consequences (to be used with caution)** | - 5.2 Use methods specifically designed to emphasise the consequences of performing the behaviour with the aim of making them more memorable (goes beyond informing about consequences) |
| **Stigma/ social judgement for using inhalers in public** | *“Asthma patients never feel comfortable to use asthma medications especially inhaler in public because by this act they feel stigmatized”* | Social influences | - 3.1 social support (unspecified) - 3.2 social support (practical) - 6.2 social comparison - 6.3 information about others' approval - 10.4 social reward | - 3.1: practicable – could suggest the app user asks a person in their social circle to speak up when others are being less supportive of inhaler use - 3.2: practicable – could suggest the app user asks a person in their social circle to speak up when others are being less supportive of inhaler use - 6.2 less relevant to this influence - 6.3 practicable but the others would need to be people whose opinions matter to the participant as much as those being critical - 10.4 less relevant for this influence | - 3.1 acceptable only if app user can identify at least one supportive person - 3.2 acceptable only if app user can identify at least one supportive person - 6.3 acceptable | - **3.1 social support (unspecified)** - **3.2 social support (practical)** - **6.3 information about others' approval [only if can identify others whose approval matters to the person as much as the less supportive individuals' opinions]** | - 3.1 Advise on, arrange or provide social support (e.g. from friends, relatives, colleagues,’ buddies’ or staff) or noncontingent praise or reward for performance of the behaviour. - 3.2 Advise on, arrange, or provide practical help (e.g. from friends, relatives, colleagues, ‘buddies’ or staff) for performance of the behaviour - 6.3 Provide information about what other people think about the behaviour. The information clarifies whether others will like, approve or disapprove of what the person is doing or will do |
| **Opinions of friends/ family/ media (doubt from others that inhaler is needed)** | *“Attitudes toward taking asthma medications are influenced by friends, family, the media”* | Subjective norms | - 6.2 social comparison - 6.3 information about others approval | - 6.2 not relevant - 6.3 possible - issue is how to identify others whose approval would counteract the doubts of friends and family | - 6.3 acceptable if can identify relevant others to provide approval | - **6.3 information about others' approval** | - 6.3 Provide information about what other people think about the behaviour. The information clarifies whether others will like, approve or disapprove of what the person is doing or will do |
| **Poor HCP/ patient communication** | *“When I go to my appointment, they try to rush, get you in there, rush you out, get you some stuff, ‘Take this, do this’”* | Social influences | - 3.1 social support (unspecified) - 3.2 social support (practical) - 6.2 social comparison - 6.3 information about others' approval - 10.4 social reward | - 3.1 could be delivered digitally as messages from a healthcare professional (HCP) - 3.2 could be delivered digitally as messages from a HCP - 6.2 less relevant to this influence - 6.3 could also be done via digital messaging from a HCP - 10.4 less relevant to this influence | - Acceptability of 3.1, 3.2 & 6.3 would depend on whether app developers could arrange communication with a HCP the app users feel they have a meaningful relationship with. | - **3.1 social support (unspecified)** - **3.2 social support (practical)** - **6.3 information about others' approval** | - 3.1 Advise on, arrange or provide social support (e.g. from friends, relatives, colleagues,’ buddies’ or staff) or noncontingent praise or reward for performance of the behaviour. - 3.2 Advise on, arrange, or provide practical help (e.g. from friends, relatives, colleagues, ‘buddies’ or staff) for performance of the behaviour - 6.3 Provide information about what other people think about the behaviour. The information clarifies whether others will like, approve or disapprove of what the person is doing or will do |
| **Lack of time to take inhalers (daily life )** | *“At least one squirt and it does something. Not take two, breathe in for 10, hold that. You haven’t got time. You really don’t.”* | Environmental context and resources | - 3.2 social support (practical) - 7.1 prompts/cues - 7.5 remove aversive stimulus - 12.1 restructuring the physical environment - 12.2 restructuring the social environment - 12.3 avoiding/reducing exposure to cues for the behaviour - 12.5 adding objects to the environment | - 3.2 practicable – could implement as asking others to remind the app user to use their inhaler - 7.1 less relevant to this influence - 7.5 not relevant - 12.1 depends on where restructuring needs to take place (work vs. home) but relevant and potentially practical - 12.3 not relevant - 12.5 less relevant to this influence | - 3.2 acceptability depends on setting - people might not be keen to ask work colleagues - 12.1 acceptability depends on location of restructuring | - **3.2 social support (practical)** - **12.1 restructuring the physical environment** | - 3.2 Advise on, arrange, or provide practical help (e.g. from friends, relatives, colleagues, ‘buddies’ or staff) for performance of the behaviour - 12.1 Change, or advise to change the physical environment in order to facilitate performance of the wanted behaviour or create barriers to the unwanted behaviour (other than prompts/cues, rewards and punishments) |
| **Inhalers out of date due to irregular use** | *“Yes, I’ve had one of them but by the time I get round to taking it they’re out of date, so…”* | Environmental context and resources | - 3.2 social support (practical) - 7.1 prompts/cues - 7.5 remove aversive stimulus - 12.1 restructuring the physical environment - 12.2 restructuring the social environment - 12.3 avoiding/reducing exposure to cues for the behaviour - 12.5 adding objects to the environment | - Practicability of all options low. The authors suggest it would be better to tackle this influence indirectly, by reducing non-adherence through other means. If people use their inhalers more regularly, they are less likely to go out of date. | - N/A | **N/A –suggest tackling this barrier by enhancing adherence by other means** |  |
| **Not wanting to identify as a ‘sick’ person** | “I hate feeling like I’m different. I have a problem when it comes to that and I hold off on my medicine” | Social /professional role and identity  possibly also self-image | **Social/professional role and identity:**  There was no expert consensus on links but there were inconclusive links made between this MoA and :   - 3.1 social support (unspecified) - 6.2 social comparison - 9.1 credible source - 13.5 identity associated with changed behaviour   **Self-image:**   - 13.1 identification of self as a role model | ***Social/professional role and identity:***   - 3.1 – possibly practicable, if can provide social support suggesting that inhaler use doesn't define the app user as a sick person - 6.2 - could be relevant, if could find role model who uses inhaler more but is likely to be perceived as healthy/fit (e.g. a sportsperson) - 9.1 relevant and practicable - 13.5 tricky to use digitally   ***Self-image:***   - 13.1 could backfire – may accidentally reinforce identity as a healthy person who does not need to use inhalers | - 3.1 acceptable - 6.2 acceptable if choose appropriate person for app users to compare themselves to - 9.1 acceptable | - **3.1 social support (unspecified)** - **6.2 social comparison** - **9.1 credible source** | - 6.2 Draw attention to others’ performance to allow comparison with the person’s own performance - 9.1 Present verbal or visual communication from a credible source in favour of or against the behaviour |
| **Level of acceptance of asthma as part of identity** | *“It depends on whether you’ve accepted asthma as part of your identity”* | Self-image | - 13.1 identification of self as a role model | - 13.1 reasonably practicable – needs app user to have relationship with someone to whom they could serve as a role model | - 13.1 potentially acceptable | - **13.1 identification of self as a role model** | - 13.1 Inform that one's own behaviour may be an example to others |
| **Not wanting to feel controlled by asthma/ dependent on medication** | *“Restricting use of asthma medicines (to not to feel controlled or dependent on medicines)”* | Possibly belief about consequences if a consequence of adherence is feeling controlled/ dependent, Self-image; | ***Belief about consequences***   - 5.1 information about health consequences - 5.2 salience of consequences - 5.3 information about social and environmental consequences - 5.5 anticipated regret - 5.6 information about emotional consequences - 9.2 pros and cons - 9.3 comparative imagining of future outcomes - 10.1 material incentive (behaviour) - 10.8 incentive (outcome) - 10.10 reward (outcome)   **Self-image:**   - 13.1 identification of self as a role model | ***Belief about consequences***   - 5.1 practicable to deliver digitally, - 5.2 practicable, - 5.3 practicable - 5.5 practicable - 5.6 relevant - 9.2 could do as a task - make your own mind up - after information provision - 9.3 tricky to do digitally - 10.xx (incentive/reward BCTs) not relevant to this influence   ***Self-image***   - 13.1 - could backfire (the person is already using their adherence behaviour to present themselves in a certain way) | ***Belief about consequences***   - 5.1 acceptable, - 5.2 acceptable, - 5.3 acceptable, - 5.5 potentially low acceptability – sometimes seen as “guilt trip” - 5.6 acceptable, - 9.2 acceptable | **Beliefs about consequences:**   - **5.1 information about health consequences** - **5.2 salience of consequences** - **5.3 information about social and environmental consequences** - **5.6 information about emotional consequences** - **9.2 pros and cons**   **Self-image**   - **N/A** | - 5.1 Provide information (e.g. written, verbal, visual) about health consequences of performing the behaviour - 5.2 Use methods specifically designed to emphasise the consequences of performing the behaviour with the aim of making them more memorable (goes beyond informing about consequences) - 5.3 Provide information (e.g. written, verbal, visual) about social and environmental consequences of performing the behaviour - 5.6 Provide information (e.g. written, verbal, visual) about emotional consequences of performing the behaviour - 9.2 Advise the person to identify and compare reasons for wanting (pros) and not wanting to (cons) change the behaviour |
| **Doubt about asthma diagnosis** | *“In terms of her diagnosis, she doesn’t believe it addressed the actual condition she had, and she still believes it doesn’t”* | General attitude/belief.  Self-image | **General attitude/belief:**   - 9.1 credible source - 9.2 pros and cons   **Self-image:**   - 13.1 identification of self as a role model | - 9.1 possible - issue is identifying the most credible source for the broadest range of users - 9.2 potentially practicable - could implement as having app users list the pros and cons of "running with the asthma diagnosis and seeing what happens [if I take my medications regularly]" - 13.1 not relevant to this type of influence | - 9.1 – acceptable only if source is perceived as credible - 9.2 potentially acceptable if presented with appropriate delivery style | **General attitude/belief:**   - **9.1 credible source** - **9.2 pros and cons**   **Self-image:**   - **N/A** | - 9.1 Present verbal or visual communication from a credible source in favour of or against the behaviour - 9.2 Advise the person to identify and compare reasons for wanting (pros) and not wanting to (cons) change the behaviour |
| **Confidence in using inhalers** | *“Medication adherence for asthma is strongly influenced by self efficacy levels”* | Beliefs about capabilities | - 1.2 problem solving - 4.1 instruction on how to perform the behaviour - 6.1 demonstration of the behaviour - 8.1 behavioural practice/rehearsal - 8.7 graded tasks - 15.1 verbal persuasion about capability - 15.3 focus on past success - 15.4 self-talk | - 1.2 - hard to do well without human input, can be demotivating if person identifies barriers but can’t find solutions - 4.1 feasible - 6.1 feasible, - 8.1 feasible - 8.7 less feasible - 15.1 feasible though not always effective - 15.3 feasible but effectiveness does depend on whether the app user has had past success - 15.4 could offer users a selection of positive things to tell themselves, but possibly a little contrived | - 4.1 acceptable - 6.1 acceptable - 8.1 acceptable - 15.1 might not be convincing unless based on some prior knowledge of the person - 15.3 acceptable to app users with some past success - 15.4 some app users may find affirmations a little embarrassing and awkward | - **4.1 instruction on how to perform the behaviour** - **6.1 demonstration of the behaviour** - **8.1 behavioural practice/rehearsal** - **15.1 verbal persuasion about capability – possible but would require some prior knowledge of the person's successful inhaler use** - **15.3 focus on past success – possible but would require some prior knowledge of the person's successful inhaler use** | - 4.1 Advise or agree on how to perform the behaviour - 6.1 Provide an observable sample of the performance of the behaviour, directly in person or indirectly e.g. via film, pictures, for the person to aspire to or imitate - 8.1 Prompt practice or rehearsal of the performance of the behaviour one or more times in a context or at a time when the performance may not be necessary, in order to increase habit and skill - 15.1 Tell the person that they can successfully perform the wanted behaviour, arguing against self-doubts and asserting that they can and will succeed - 15.3 Advise to think about or list previous successes in performing the behaviour (or parts of it) |
| **Perceived efficacy of inhalers** | *“Adherence is positively affected by the belief that giving inhaled corticosteroids would protect the patient from getting worse”* | Beliefs about consequences | - 5.1 information about health consequences - 5.2 salience of consequences - 5.3 information about social and environmental consequences - 5.5 anticipated regret - 5.6 information about emotional consequences - 9.2 pros and cons - 9.3 comparative imagining of future outcomes - 10.1 material incentive (behaviour) - 10.8 incentive (outcome) - 10.10 reward (outcome) | - 5.1 practicable, - 5.2 practicable. - 5.3 - practicable - 5.5 - less relevant – the person has to believe an inhaler's effective to anticipate regretting not using it - 5.6 practicable - 9.2 less relevant to this influence - 9.3 less relevant to this influence - 10.1 – not relevant for this influence - 10.8 – not relevant for this influence - 10.10 – not relevant for this influence | - 5.1 - acceptable, - 5.2 – acceptable - 5.3 – acceptable - 5.6 - acceptable | - **5.1 information about health consequences** - **5.2 salience of consequences** - **5.3 information about social and environmental consequences** - **5.6 information about emotional consequences** | - 5.1 Provide information (e.g. written, verbal, visual) about health consequences of performing the behaviour - 5.2 Use methods specifically designed to emphasise the consequences of performing the behaviour with the aim of making them more memorable (goes beyond informing about consequences) - 5.3 Provide information (e.g. written, verbal, visual) about social and environmental consequences of performing the behaviour - 5.6 Provide information (e.g. written, verbal, visual) about emotional consequences of performing the behaviour |
| **Beliefs about side effects** | *“At least 50% refused to adhere to their prescribed therapy fully because of concerns over side effects”* | Beliefs about consequences | - 5.1 information about health consequences - 5.2 salience of consequences - 5.3 information about social and environmental consequences - 5.5 anticipated regret - 5.6 information about emotional consequences - 9.2 pros and cons - 9.3 comparative imagining of future outcomes - 10.1 material incentive (behaviour) - 10.8 incentive (outcome) - 10.10 reward (outcome) | - 5.1 practicable - 5.2 feasible to deliver - 5.3 less relevant to this influence - 5.5 hard to apply well to this influence - side effects concerns are often tied to anticipated regret at using the drug - 5.6 less relevant to this influence - 9.2 – practicable, but to be effective may need to first provide information to alter perceived pros and cons - 9.3 – risk that if implemented without a human intervention facilitator, this BCT could make potential adverse effects more vivid, so decreasing adherence - 10.1 - incentives unlikely to overcome concerns - 10.8 - incentives unlikely to overcome concerns - 10.10 potentially feasible, depending on nature of reward. | - 5.1 acceptable, as long as tone is not overly didactic, acknowledges potential side effects do exist - 5.2 – acceptable, as long as tone is not overly didactic, acknowledges potential side effects do exist - 9.2 acceptable - 10.10 less acceptable – risk app users may perceive reward as a bribe to overcome their legitimate concerns | - **5.1 information about health consequences** - **5.2 salience of consequences possibly** - **9.2 pros and cons** | - 5.1 Provide information (e.g. written, verbal, visual) about health consequences of performing the behaviour - 5.2 Use methods specifically designed to emphasise the consequences of performing the behaviour with the aim of making them more memorable (goes beyond informing about consequences) - 9.2 Advise the person to identify and compare reasons for wanting (pros) and not wanting to (cons) change the behaviour |
| **Concern that inhalers do more harm than good** | *“Barriers related to these facts included the wrong beliefs; for instance, asthma medicines may be more harmful than beneficial”* | Beliefs about consequences | - 5.1 information about health consequences - 5.2 salience of consequences - 5.3 information about social and environmental consequences - 5.5 anticipated regret - 5.6 information about emotional consequences - 9.2 pros and cons - 9.3 comparative imagining of future outcomes - 10.1 material incentive (behaviour) - 10.8 incentive (outcome) - 10.10 reward (outcome) | - 5.1 practicable - 5.2 - practicable. - 5.3 less relevant to this influence - 5.5 hard to apply well to this belief - harm concerns are often tied to anticipated regret at using the drug - 5.6 less relevant to this influence - 9.2 - practicable, but to be effective may need to first provide information to alter perceived pros and cons - 9.3 risk that could push adherence in the wrong direction - 10.1, 10.8 - incentives unlikely to overcome concerns - 10.10 hard to arrange | - 5.1 – acceptable but tone important - not overly didactic, acknowledge potential side effects do exist and people may have prior experience with them. - 5.2 – acceptable but tone important - not overly didactic, acknowledge potential side effects do exist and people may have prior experience with them - 9.2 likely to be acceptable | - **5.1 information about health consequences** - **5.2 salience of consequences** - **9.2 pros and cons** | - 5.1 Provide information (e.g. written, verbal, visual) about health consequences of performing the behaviour - 5.2 Use methods specifically designed to emphasise the consequences of performing the behaviour with the aim of making them more memorable (goes beyond informing about consequences) - 9.2 Advise the person to identify and compare reasons for wanting (pros) and not wanting to (cons) change the behaviour |
| **Perceiving cause and effect of using inhalers on asthma symptoms** | *“I’ve had times when I have been on strict medication regimes and I have still had attacks”* | Beliefs about consequences | - 5.1 information about health consequences - 5.2 salience of consequences - 5.3 information about social and environmental consequences - 5.5 anticipated regret - 5.6 information about emotional consequences - 9.2 pros and cons - 9.3 comparative imagining of future outcomes - 10.1 material incentive (behaviour) - 10.8 incentive (outcome) - 10.10 reward (outcome) | - 5.1 practicable - 5.2 practicable - 5.3 not relevant to this influence - 5.5 not relevant to this influence - 5.6 not relevant to this influence - 9.2 not relevant - 9.3 less relevant to this influence - 10.1 not relevant, 10.8 not relevant, 10.10 not relevant | - 5.1 likely to be acceptable if pitched at appropriate level - 5.2 likely to be acceptable if pitched at appropriate level l | - **5.1 information about health consequences** - **5.2 salience of consequences** | - 5.1 Provide information (e.g. written, verbal, visual) about health consequences of performing the behaviour - 5.2 Use methods specifically designed to emphasise the consequences of performing the behaviour with the aim of making them more memorable (goes beyond informing about consequences) |
| **Embarrassment (e.g. of using inhalers in public)** | *“They identified some particular issues such as …embarrassment over medication use in public among asthma patients”* | Beliefs about consequences  Emotions | - 5.1 information about health consequences - 5.2 salience of consequences - 5.3 information about social and environmental consequences - 5.5 anticipated regret - 5.6 information about emotional consequences - 9.2 pros and cons - 9.3 comparative imagining of future outcomes - 10.1 material incentive (behaviour) - 10.8 incentive (outcome) - 10.10 reward (outcome)   **Emotions**   - 11.2 reduce negative emotions | **Beliefs about consequences**   - 5.1 not relevant to this particular influence - 5.2 practicable - 5.3 possible - 5.5 possible - 5.6 practicable - 9.2 could work as digital listing task - 9.3 tricky to do digitally and may backfire – if imagined embarrassment is higher than imagined benefits, - 10.1 incentives less relevant to this particular influence - 10.8: incentives less relevant to this particular influence - 10.10: rewards less relevant to this particular influence   **Emotions:**   - 11.2 practicable | **Beliefs about consequences**   - 5.2 acceptable, - 5.3 acceptable, - 5.5 less acceptable – may be perceived as "guilt trip," - 9.2 acceptable   **Emotions**   - 11.2 acceptable | **Beliefs about consequences:**   - **5.2 salience of consequences** - **5.3 information about social and environmental consequences** - **5.6 information about emotional consequences** - **9.2 pros and cons**   **Emotions**   - **11.2 Advise on ways of reducing negative emotions to facilitate performance of the behaviour** | **Beliefs about consequences**   - 5.2 Use methods specifically designed to emphasise the consequences of performing the behaviour with the aim of making them more memorable (goes beyond informing about consequences) - 5.3 Provide information (e.g. written, verbal, visual) about social and environmental consequences of performing the behaviour - 5.6 Provide information (e.g. written, verbal, visual) about emotional consequences of performing the behaviour - 9.2 Advise the person to identify and compare reasons for wanting (pros) and not wanting to (cons) change the behaviour   **Emotions**   - 11.2 Advise on ways of reducing negative emotions to facilitate performance of the behaviour |
| **Discomfort with long term use of inhalers** | *“one factor in their lack of adherence was high discomfort with long-term use of ICS”* | Beliefs about consequences  Emotions | - 5.1 information about health consequences - 5.2 salience of consequences - 5.3 information about social and environmental consequences - 5.5 anticipated regret - 5.6 information about emotional consequences - 9.2 pros and cons - 9.3 comparative imagining of future outcomes - 10.1 material incentive (behaviour) - 10.8 incentive (outcome) - 10.10 reward (outcome)   **Emotions**   - 11.2 reduce negative emotions | - 5.1 practicable, - 5.2 practicable, - 5.3 less relevant to this influence - 5.5 less relevant to this influence, - 5.6 practicable and relevant if frame emotional consequences as reassurance about not having severe asthma attacks - 9.2 practicable but may want to combine with some information provision (e.g. BCTs 5.1, 5.2, 5.6) to maximise impact on adherence - 10:1 incentives less relevant to this influence - 10.8 incentives less relevant to this influence - 10.10 rewards less relevant to this influence - 11.2 practicable | - 5.1 acceptable, - 5.2 acceptable, - 5.6 potentially acceptable, - 9.2 acceptable - 11.2 potentially acceptable but would need to be done sensitively – not invalidating person’s emotions | - **5.1 information about health consequences** - **5.2 salience of consequences** - **5.6 information about emotional consequences** - **9.2 pros and cons** - **11.2 reduce negative emotions** | - 5.1 Provide information (e.g. written, verbal, visual) about health consequences of performing the behaviour - 5.2 Use methods specifically designed to emphasise the consequences of performing the behaviour with the aim of making them more memorable (goes beyond informing about consequences)6 Provide information (e.g. written, verbal, visual) about emotional consequences of performing the behaviour - 9.2 Advise the person to identify and compare reasons for wanting (pros) and not wanting to (cons) change the behaviour   **Emotions**   - 11.2 Advise on ways of reducing negative emotions to facilitate performance of the behaviour |
| **Prioritising asthma to support** | *“I’ve got 2 kids; I cannot afford to be sick, I cannot afford not to have the energy to work and to do all of these things. So, I will take it"* | Goals | - 1.1 goal setting (behaviour) - 1.3 goal setting (outcome) - 1.5 review behaviour goals - 1.6 discrepancy between current behaviour and goal - 1.7 review outcome goal(s) | - 1.1 not relevant to this influence - 1.3 possible – if implemented as some sort of broad life goal setting activity with a prioritisation element - 1.5 not relevant to this influence - 1.6 not relevant - 1.7 feasible but would require monitoring of outcome goal achievements first to be able to implement this | - 1.3 potentially acceptable, - 1.7 potentially acceptable, but high burden on app users to self monitor | - **1.3 goal setting (outcome)** | - 1.3 Set or agree on a goal defined in terms of a positive outcome of wanted behaviour |

^a^ The numbering for each BCT is the numeric code used to refer to each one in the BCT Taxonomy v1 (Michie et al, 2013).

^b^ Acceptability was only considered for BCTs that were judged practicable to deliver

* BCTs were judged as “not relevant to this influence” where the BCT was unlikely to change the specific influence on adherence. The Theory and Techniques (TaT) Tool pairs BCTs with broad categories of mechanisms of action (e.g. “beliefs about consequences”). However, the influences on preventer adherence behaviour are narrower than these broad categories and therefore not all BCTs suggested for a broad category are relevant for a specific influence.
